# Supplementary material for: The genome sequence of the Lilac Beauty, Apeira syringaria (Linnaeus, 1758)
Source: Wellcome Open Res. 2023 Apr 12;8:159. [Version 1] doi: 10.12688/wellcomeopenres.19208.1 (PMC11128049; doi:10.12688/wellcomeopenres.19208.1)
Supplement: Peer review history

# Reviewer report: Reviewer response for version 1

**DOI:** 10.21956/wellcomeopenres.21290.r70920

**Published:** 2024-05-25

**Authors:** Violaine Llaurens

**Affiliations:** 1 Institut de Systématique, Evolution et Biodiversité, Museum National d'Histoire Naturelle, Paris, Île-de-France, France

The article carefully describes the sequencing, assembly and annotation of the genome of a lilac beauty female, following a state-of-the-art workflow. I have only two questions 

1. Did the author specifically search for the W chromosome?
2. In figure 5, we can clearly see that one of the scaffold does not seem to have any contact with the others. This is quite unusual to me. Is there any biological and/or technical reason that my explain this uncommon feature of the Hi-C map?

Are sufficient details of methods and materials provided to allow replication by others?

Yes

Is the rationale for creating the dataset(s) clearly described?

Yes

Are the datasets clearly presented in a useable and accessible format?

Yes

Are the protocols appropriate and is the work technically sound?

Yes

Reviewer Expertise:

evolutionary ecology, population genomics

I confirm that I have read this submission and believe that I have an appropriate level of expertise to confirm that it is of an acceptable scientific standard.

# Reviewer report: Reviewer response for version 1

**DOI:** 10.21956/wellcomeopenres.21290.r69167

**Published:** 2023-11-09

**Authors:** Jurate De Prins

**Affiliations:** 1 Royal Belgian Institute of Natural Sciences, Brussels, Belgium

My review is only from the taxonomic point of view, for molecular details please apply to a molecular specialist. From the taxonomic point of view, this article looks ok. Just a couple of small details:

- I would suggest to explain in more detail what the authors mean by "an unusual resting posture". What is usual then in Geometridae? Geometridae moths, differently from the majority of other moths, rest with open wings.
- It would be good to mention that *Apeira syringaria* (Linnaeus, 1758) is widely distributed in Europe.

Are sufficient details of methods and materials provided to allow replication by others?

Yes

Is the rationale for creating the dataset(s) clearly described?

Yes

Are the datasets clearly presented in a useable and accessible format?

Yes

Are the protocols appropriate and is the work technically sound?

Yes

Reviewer Expertise:

Systematics, biodiversity, taxonomy, bioinformatics, Lepidoptera

I confirm that I have read this submission and believe that I have an appropriate level of expertise to confirm that it is of an acceptable scientific standard.
